# REST: A Toolkit for Resting-State Functional Magnetic Resonance Imaging Data Processing

**DOI:** 10.1371/journal.pone.0025031

**Published:** 2011-09-20

**Authors:** Xiao-Wei Song, Zhang-Ye Dong, Xiang-Yu Long, Su-Fang Li, Xi-Nian Zuo, Chao-Zhe Zhu, Yong He, Chao-Gan Yan, Yu-Feng Zang

**Affiliations:** 1 State Key Laboratory of Cognitive Neuroscience and Learning, Beijing Normal University, Beijing, China; 2 Institute of Biophysics, Chinese Academy of Sciences, Beijing, China; 3 Institute of Psychology, Chinese Academy of Sciences, Beijing, China; 4 Center for Cognition and Brain Disorders and The Affiliated Hospital, Hangzhou Normal University, Hangzhou, Zhejiang, China; The University of Melbourne, Australia

## Abstract

Resting-state fMRI (RS-fMRI) has been drawing more and more attention in recent years. However, a publicly available, systematically integrated and easy-to-use tool for RS-fMRI data processing is still lacking. We developed a toolkit for the analysis of RS-fMRI data, namely the RESting-state fMRI data analysis Toolkit (REST). REST was developed in MATLAB with graphical user interface (GUI). After data preprocessing with SPM or AFNI, a few analytic methods can be performed in REST, including functional connectivity analysis based on linear correlation, regional homogeneity, amplitude of low frequency fluctuation (ALFF), and fractional ALFF. A few additional functions were implemented in REST, including a DICOM sorter, linear trend removal, bandpass filtering, time course extraction, regression of covariates, image calculator, statistical analysis, and slice viewer (for result visualization, multiple comparison correction, etc.). REST is an open-source package and is freely available at http://www.restfmri.net.

## Introduction

Most functional magnetic resonance imaging (fMRI) studies focus on task-state conditions. However, since the first paper of resting-state fMRI (RS-fMRI) [1], RS-fMRI has been drawing more and more attention, especially in recent years [2]. A few studies have indicated that the RS-fMRI signal, i.e., the resting-state blood oxygenation level dependent (BOLD) signal, reflects spontaneous brain activity [3]–[5]. Compared with resting-state position emission tomography (PET) and single photon emission computerized tomography (SPECT), RS-fMRI has no ionizing radiation. Compared with task-state fMRI, RS-fMRI is easier to perform for both the investigators and participants. Together with recent studies showing promising reliability for RS-fMRI [6]–[8], the evidence indicates that RS-fMRI may be very helpful in clinical studies. However, data processing toolkit specialized for RS-fMRI is still lacking. The current paper would introduce a novel and open source software package.

The analytical approaches for RS-fMRI can be divided into two categories. One is for measuring the functional relationship between distinct brain regions. This procedure is usually referred to as functional connectivity analysis, for which linear correlation [1] and independent component analysis (ICA) [9] are two frequently used methods. Several toolkits have been proposed for functional connectivity analysis, including the Group ICA of fMRI Toolbox (GIFT; http://icatb.sourceforge.net/groupica.htm) [10], Multivariate Exploratory Linear Optimized Decomposition into Independent Components (MELODIC) [11], the MATLAB Toolbox for Functional Connectivity [12], and the Functional Connectivity Toolbox (http://web.mit.edu/swg/software.htm). Functional connectivity measures the similarity or synchronization of the time courses between distinct brain regions. However, for clinical studies, a result of abnormal functional connectivity between two or more distinct brain regions could not provide direct information as to which brain region is abnormal, as functional connectivity assesses only the relationship between them.

Another category of analytical approaches for RS-fMRI is based on depicting local features of the spontaneous BOLD signal. Two examples are the measurements of regional homogeneity (ReHo) [13] and the amplitude of low frequency fluctuation (ALFF) [1], [14]. By calculating the Kendall coefficient of concordance (KCC), ReHo measures the similarity or synchronization of the time courses within a cluster (e.g., 27 neighboring voxels) [13]. For each single voxel, ALFF measures the magnitude of the fluctuation of the voxel [14]. ReHo and ALFF have been used to study brain disorders including attention deficit hyperactivity disorder (ADHD) [14], [15], schizophrenia [16]–[18], depression [19], [20], Alzheimer's disease (AD) [21]–[23], mild cognitive impairment [24], Parkinson disease (PD) [25], epilepsy [26], and posttraumatic stress disorder [27]. Accurate localization of the abnormal spontaneous brain activity is crucial in the clinical treatment of some specific diseases, e.g., focal resection of intractable epilepsy and deep brain stimulation (DBS) for PD and depression. To this end, a convenient toolkit is necessary and important for the analysis of RS-fMRI data for basic and clinical neuroscientists.

Previously, most procedures for data preprocessing and group level statistical analysis have been well implemented in freely available analysis packages such as Statistical Parametric Mapping (SPM) [28], Analysis of Functional NeuroImages (AFNI) [29], and the FMRIB Software Library (FSL) [30]. Meanwhile, there exist implementations of RS-fMRI data analysis combining various commands from AFNI and FSL without an integrated graphical user interface (GUI), among which fcon_1000_script integrates both seed-based and ICA-based functional connectivity analyses and is publicly available [2].

Here we present a MATLAB package named REST (*RESting-state fMRI data analysis Toolkit*). It represents a publicly available, systematically integrated, and ease-to-use implementation of various RS-fMRI analyses. The software configures various parameters for data analysis easily and quickly with an integrated GUI and also supports batch jobs. Based on MATLAB, REST can exchange files/data with SPM, AFNI, and FSL under the NIfTI-1.1 or ANALYZE™ 7.5 formats. Two measures of local features for RS-fMRI signal analysis, ReHo and ALFF, were implemented as core features in the current version of REST. Given that the Pearson linear correlation is a widely used method in RS-fMRI analysis, it was also implemented in REST. Some additional functions were implemented in REST, including DICOM sorter, linear trend removal, bandpass filtering, regression of covariates, time course extraction, statistical analysis, image calculator, and slice viewer (for result visualization, multiple comparison correction, etc.).

## Results

### 1. Algorithms

In this section, we introduce the three main algorithms in REST: functional connectivity, ReHo, and ALFF.

#### 1.1. Functional connectivity

Since the seminal RS-fMRI study by [1], the Pearson linear correlation has been the most widely used algorithm to measure the functional connectivity in RS-fMRI studies. REST also uses the Pearson correlation to calculate functional connectivity between two time courses:
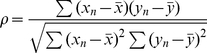
(1)where 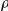 is the Pearson correlation coefficient, 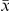 and 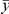 are the mean values of time course x and y, respectively.

In the literature [31], some covariates are usually regressed out before functional connectivity analysis, including the time courses of head motion (three for translation and three for rotation), mean time course of the entire brain (or global trend), white matter, and cerebrospinal fluid (CSF). REST supports these regression procedures with some predefined (user-defined or default) masks. The default masks in REST were made from the *a priori* templates found in SPM (http://www.fil.ion.ucl.ac.uk/spm) as follows: the whole brain mask (named BrainMask_05_61x73x61.img/hdr) was from brainmask.nii with a threshold at 50% probability, the white matter mask (named WhiteMask_09_61x73x61.img/hdr) was from white.nii with a threshold at 90% probability, and the CSF mask (named CsfMask_07_61x73x61.img/hdr) was from csf.nii with a threshold at 70% probability. Partial correlation will be performed if the aforementioned covariates are added. It should be noted that removal of the global mean signal is still controversial in RS-fMRI [32], [33]. It has been proposed that the anti-correlation which has been widely reported (e.g., [31]) was most likely due to the removal of the global mean signal [33].

#### 1.2. Regional homogeneity (ReHo)

The computation of ReHo in REST has been previously described in [13]. Briefly, Kendall's coefficient of concordance (KCC) for the time course of a voxel with those of its nearest neighbors (6, 18, and 26, respectively) is calculated in a voxel-wise way as follows:
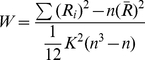
(2)where W is the KCC of a cluster, ranging from 0 to 1; 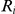 is the sum rank of the *i*th time point; 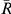 = (K(n+1))/2 is the mean of the 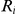
's; *K* is the number of time courses within a measured cluster (here, K = 7, 19, and 27, respectively; one chosen voxel plus the number of its neighbors); and *n* is the number of ranks. From this equation, an individual ReHo map is obtained. For PET studies, an individual image is usually divided by the global mean value to minimize inter-individual variability [34]. REST also includes this normalization procedure in the ReHo calculation. In the other words, an individual ReHo map could be divided by the mean ReHo value of voxels within a specific mask (whole brain mask or user-defined mask).

#### 1.3. Amplitude of low frequency fluctuations (ALFF)

Many studies have suggested that the low frequency fluctuations found in RS-fMRI BOLD signals contain physiologically meaningful information and are closely related to spontaneous neuronal activities [1], [3], [35]–[37]. On the basis of the root mean square in the paper [1], Zang et. al. [14] used the average of the amplitude of the low frequency band (i.e., ALFF) as an index of magnitude for the spontaneous neuronal activity.

ALFF is defined as the mean square root of the power spectrum density over the low frequency band (usually 0.01∼0.08 Hz). After an individual ALFF map is obtained, a standardization procedure can be performed as done in the ReHo analysis, i.e., the individual ALFF map is divided by the mean ALFF value of voxels within a specific mask (whole brain mask or user-defined mask).

It has been suggested that ALFF reflects the intensity of regional spontaneous brain activity. However, it is sensitive to the physiological noise found in some brain regions, such as in the cistern areas [14]. An improved ALFF approach, namely, fractional ALFF (fALFF) [38], is implemented in REST. fALFF is calculated as the ratio of the power spectrum of low-frequency signals (usually 0.01∼0.08 Hz) to that of the entire frequency range (e.g., 0∼0.25 Hz for TR = 2 s). This procedure has been used in near infrared spectroscopy study [39]. fALFF could significantly suppress the high frequency noise found in the cistern areas. It has been shown that the brain areas within the default mode network, including the posterior cingulate cortex (PCC), precuneus, medial prefrontal cortex (MPFC), and bilateral inferior parietal lobule (IPL), have significantly higher fALFF than the global mean fALFF [38].

Users can also choose specific frequency bands for analysis. A recent study found that the amplitude over a frequency band of 0.027∼0.073 Hz is more specific to the basal ganglia than other frequency bands [7]. It was found that the decreased ALFF in amnestic mild cognitive impairment was more prominent in a frequency band of 0.01∼0.027 Hz [40].

### 2. Usage

Installation of REST is quite easy: download the most recent version from http://www.restfmri.net, extract the compressed package to a predefined directory, and then add the full path to MATLAB's search path. REST is compatible with MATLAB version 6.5 or higher. And REST's compatibility has been tested under Windows Xp and Fedora 4. Almost all REST functions only need MATLAB basic run time environment.

**Figure 1:**
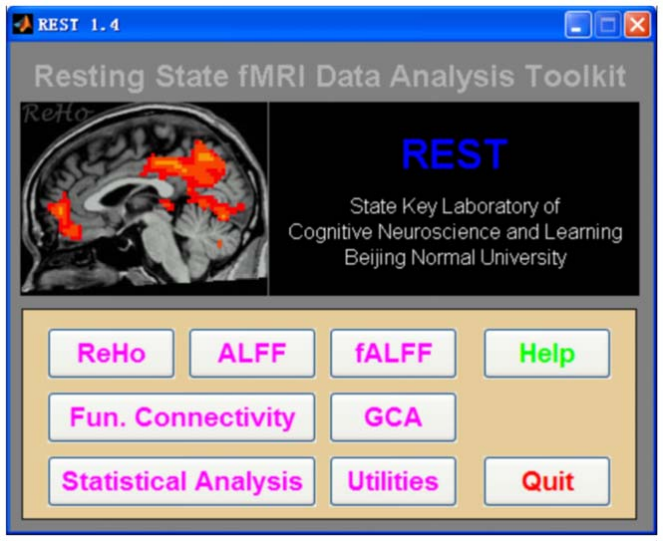
The main interface of REST.

Entering “rest” in the MATLAB command window will open REST's GUI (Fig. 1). The purple buttons seen in the GUI window allow access to the methods available for RS-fMRI data analysis. The “Help” button in REST will guide users to the RS-fMRI online forum (http://forum.restfmri.net) for more detailed information.

#### 2.1. Functional connectivity

**Figure 2:**
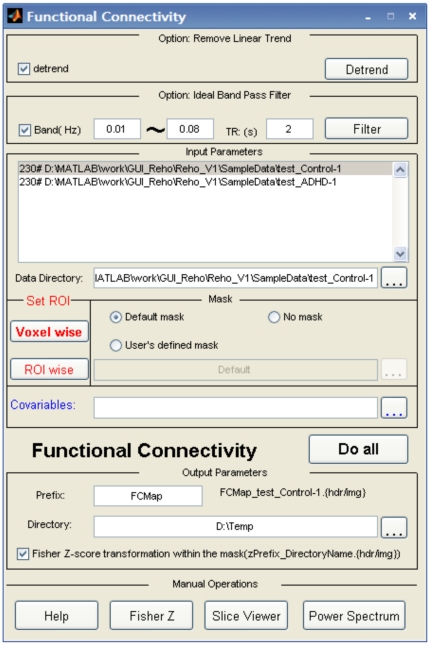
A demonstration of functional connectivity analysis. Two datasets were added. The linear trend removal and band-pass (0.01 Hz∼0.08 Hz) filtering options were then checked. A seed ROI will be set by clicking the “Voxel wise” button. The “Default mask” selection means that REST will automatically select the default mask. The usage of “Covariates” is not shown here.

Functional connectivity computation requires the user to define an ROI and optional covariates (such as the time courses of head motion, global mean, white matter, and CSF) as seen in the middle part of Fig. 2.

After the user clicks the “Fun. Connectivity” button in Fig. 1, Fig. 2 will be displayed. The button marked “Voxel wise” allows the user to define parameters for calculating the linear correlation between a reference time course (from an ROI or elsewhere) and the time course of each voxel within the entire brain or within a predefined mask. The “ROI wise” button allows the definition of parameters for calculating the linear correlation between several ROIs, and pair-wise linear correlations will be calculated. The “Covariables” button allows the user to add covariates in a text file format.

If the option “Fisher Z-score transformation within the mask” is checked, the *z* map, in addition to the *r* map, will be generated.

#### 2.2. ReHo

**Figure 3:**
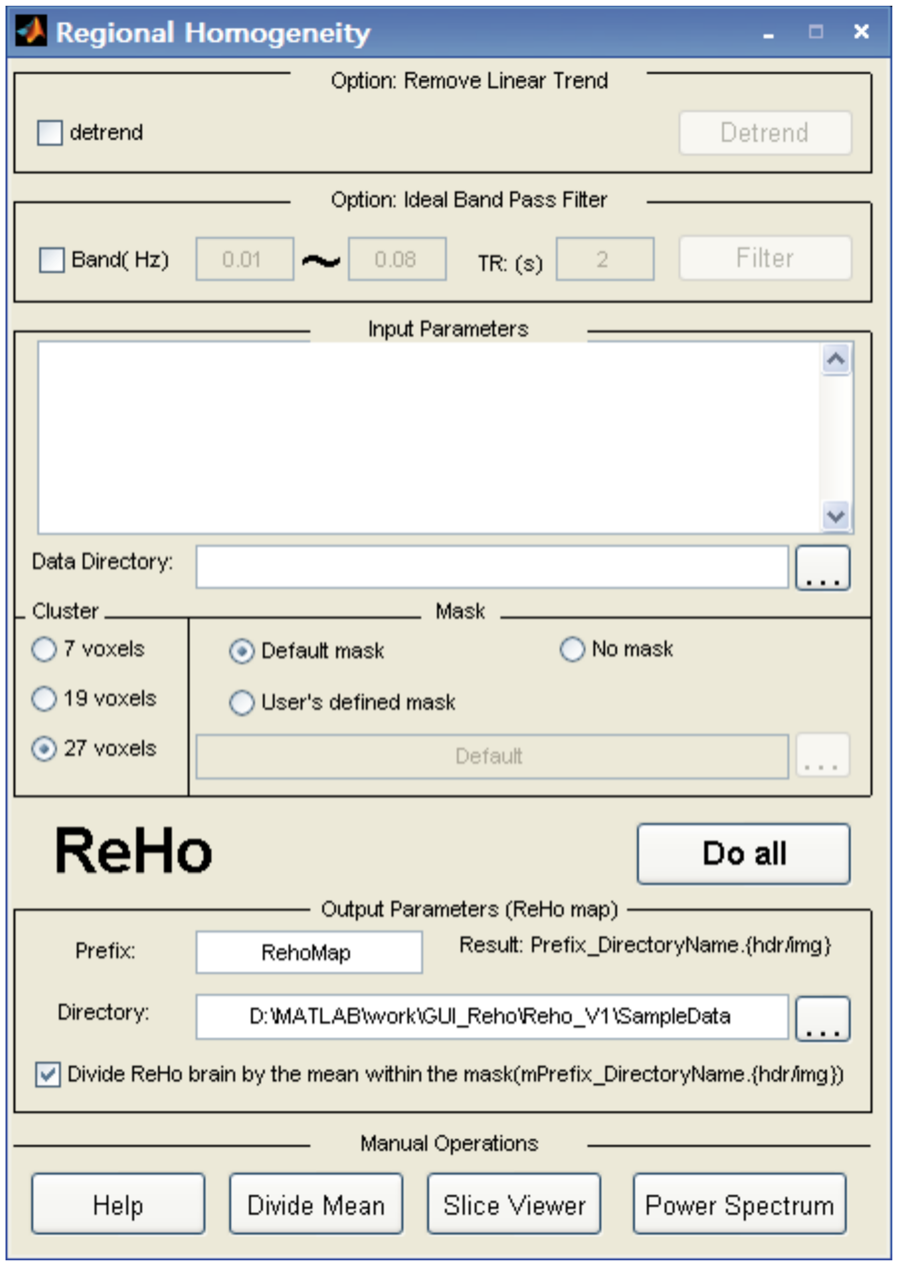
The ReHo computation GUI of REST.

After the user selects the “ReHo” button in the main interface (Fig. 1), the window illustrated in Fig. 3 will be available for ReHo analysis. In the center of the window, “cluster size” refers to the number of nearest neighbor voxels (7, 19, or 27) to be used in the analysis. In the lower part of the window, the option to “Divide ReHo brain by the mean within the mask…” will generate results with this additional data. Other parameters in the interface are similar to those found in the functional connectivity analysis.

#### 2.3. ALFF and fALFF

**Figure 4:**
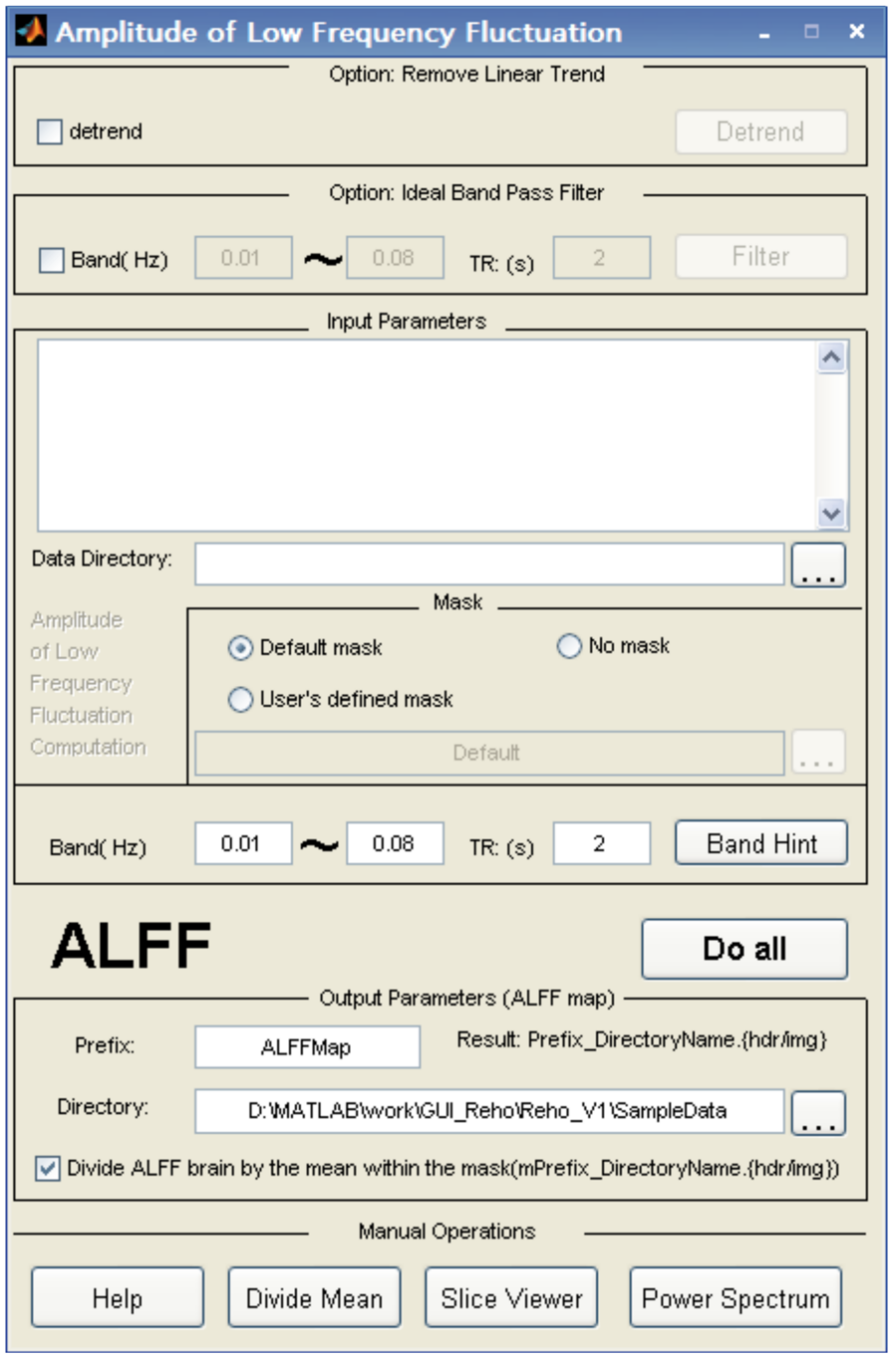
The ALFF computation GUI of REST.

After the user clicks on the “ALFF” button in the main interface (Fig. 1), the window found in Fig. 4 will come up for ALFF analysis. To transform time domain data to the frequency domain, REST uses the *fft* (MATLAB version below 7.3) or *fftw* (MATLAB version 7.3 or higher) function found in MATLAB. The amount of zero padding for the FFT is the same as the amount used in AFNI's power spectrum calculation with the option “shortest FFT length”. This specific zero padding can avoid default FFT length variability across MATLAB versions. Other parameters in the interface are similar to those in the ReHo analysis. The calculation of fALFF is similar to that of ALFF, although bandpass filtering should not be performed. The fraction of the amplitude at low frequency (0.01∼0.08) to that of the full frequency spectrum is calculated.

### 3. An example of RS-fMRI data analysis using REST

#### 3.1. Participants and data acquisition

Twenty right-handed participants (11 males, aged 19–22 years) with no history of neurological or psychiatric disorders took part in this study. All participants were recruited from the campus of Beijing Normal University. The present study was approved by the Ethics Committee of State Key Laboratory of Cognitive Neuroscience and Learning, Beijing Normal University. Written informed consent was obtained from each participant.

MRI data were acquired using a SIEMENS TRIO 3-Tesla scanner in the Imaging Center for Brain Research, Beijing Normal University. Participants lay supine with their heads snugly fixed by a belt and foam pads to minimize head movement. The functional images were obtained by using an echo-planar imaging sequence with the following parameters: 33 axial slices, slice thickness/gap = 3/0.6 mm, in-plane resolution = 64×64, TR = 2000 ms, TE = 30 ms, flip angle = 90°, FOV = 200×200 mm^2^. In addition, a three-dimensional T1-weighted magnetization-prepared rapid gradient echo (MPRAGE) sagittal image was acquired covering the entire brain (128 slices, TR = 2530 ms, TE = 3.39 ms, slice thickness = 1.33 mm, flip angle = 7°, inversion time = 1100 ms, FOV = 256×256 mm, in-plane resolution = 256×192). During the fMRI scanning, participants were instructed to keep as motionless as possible and to not think systematically. After the scanning session, all participants reported that they did not fall asleep during scanning.

#### 3.2. Data preprocessing

The first ten volumes of each functional time course were discarded to allow for T1 equilibrium and to allow the participants to adapt. Slice timing, head motion correction, and spatial normalization were conducted by using SPM5 (http://www.fil.ion.ucl.ac.uk/spm). No participant had head motion with more than 2.0 mm maximum displacement in any direction or 2.0° of any angular motion throughout the course of the scan. REST was then used for linear trend removal and temporal band-pass filtering (0.01∼0.08 Hz) [1], [41].

#### 3.3. Functional connectivity analysis

The preprocessed data were spatially smoothed (full width at half maximum (FWHM) = 6 mm) by SPM5. In the REST window for functional connectivity analysis, a spherical region of interest (ROI) (radius = 10 mm) was defined, centered at a coordinate (0, −56, 30) within the PCC [42]. For each participant, the mean time course within this ROI was used as the reference time course. A seed correlation analysis was then performed in a voxel-wise manner with the averaged time courses of the whole brain, the white matter, the CSF, and the six head motion parameters as covariates. Individual r-maps were normalized to Z-maps by using Fisher's Z transformation [43]. All Fisher's Z-maps were entered into a two-sided one-sample t-test to detect the regions showing significant functional connectivity with the PCC.

#### 3.4. ReHo analysis

In this procedure, 27 nearest neighboring voxels were defined as a cluster and a KCC value (ranging from 0 to 1) was given to the voxel at the center of this cluster [13]. In the window controlling ReHo analysis for REST, the individual ReHo map was generated and divided by that participant's global mean KCC value within the brain mask for standardization purpose. The ReHo maps were then spatially smoothed (FWHM = 6 mm) using SPM5. One-sided one-sample t-tests were performed to show where in the brain the standardized KCC value was significantly larger than one.

#### 3.5. ALFF analysis and fALFF

The preprocessed data were spatially smoothed (FWHM = 6 mm) by SPM5 before ALFF calculation. In the window for ALFF analysis within REST, the frequency band between 0.01 to 0.08 Hz was selected, and the individual ALFF map was generated [14]. The individual ALFF map was normalized by the individual's global mean ALFF as done in the ReHo analysis. One-sided one-sample t-tests were performed to show where in the brain the standardized ALFF value was significantly larger than one. The calculation of fALFF was similar to that of ALFF, but bandpass filtering was not performed. The fraction of the amplitude of low frequency signals (0.01∼0.08) to that of the full frequency spectrum was calculated.

#### 3.6. Statistical analysis

One-sample t-tests were performed on the above functional connectivity Z-maps, ReHo maps, ALFF maps, and fALFF maps, respectively, by using the “*Statistical Analysis*” methodology as implemented in REST (See “Additional Functions”). The statistical result of each method was corrected for multiple comparisons using the “AlphaSim” implementation in REST (See “Slice Viewer” in “Additional Functions”). This function is based on the Monte Carlo simulation in AFNI (see the AlphaSim command description at http://afni.nimh.nih.gov/afni/doc/manual/AlphaSim). A combination threshold of voxels' P<0.001 and cluster size >351 mm^3^ was considered significant, which corresponded with a corrected P<0.05. All within-group statistical maps obtained from the above steps were superimposed on the anatomical template (Ch2.nii) originally found in MRIcro (http://www.cabiatl.com/mricro/) by using the “Slice Viewer” routine in REST for presentation purpose.

#### 3.7. Primary Results of the Example

**Figure 5:**
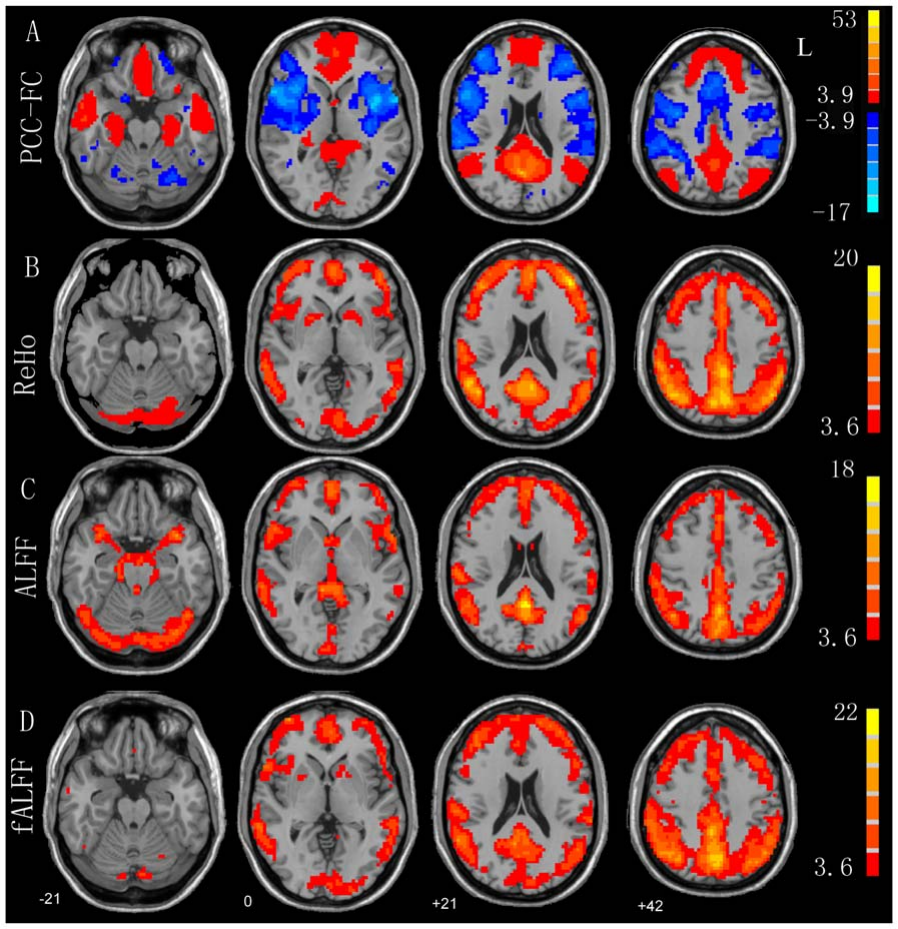
Results of one-sample t-tests (P<0.05, corrected). (A) Functional connectivity related to an ROI in the posterior cingulate cortex (PCC-FC), (B) ReHo, (C) ALFF, and (D) fALFF.

As shown in Fig. 5, some brain areas including PCC, MPFC, and bilateral IPL showed significantly higher ReHo or ALFF than the global mean. Functional connectivity analysis showed that the PCC had significant positive functional connectivity with the default mode network and significant negative functional connectivity with the attention network. These results for functional connectivity, ReHo, ALFF, and fALFF were consistent with those reported in previous studies [7], [14], [31], [38], [42], [44], [45].

### 4. Additional functions

#### 4.1. REST DICOM Sorter

Most MRI scanners produce data in Digital Imaging and Communications in Medicine (DICOM) format. The “REST DICOM Sorter” reads DICOM files by using existing routines (dicominfo and dicomread) in the MATLAB distribution and then classifies the DICOM files participant by participant and session by session. There is an option to anonymize the participants' private information (name, ID, and birthday) within the DICOM files.

#### 4.2. NIfTI.nii to NIfTI. Pairs

This routine is for converting single NIfTI.nii (3D or 4D) file(s) to NIfTI 3D pair(s) (.hdr/.img) for the convenience of FSL users. This utility supports batch mode.

#### 4.3. Reslice Image

This routine is for reslicing images to a new voxel size and new bounding box. There are several options to keep the original space or to define the new target space with an input image. The new voxel size is specified by a 1×3 matrix (such as [3 3 3]). The interpolation method can be specified by 0, 1, 2, …, n (0: Nearest Neighbor. 1: Trilinear. 2, …, n: 2nd, …, nth degree B-spline). The nearest neighbor is applied on the mask file (only integer). The routine is based on SPM (http://www.fil.ion.ucl.ac.uk/spm/).

#### 4.4. Detrend

Previous studies have demonstrated that systematic signal increase or decrease occurs over time due to long-term physiological shifts, movement related noise remaining after realignment or instrumental instability [46], [47]. The linear trend is usually removed in fMRI data preprocessing. In REST, the linear trend is estimated with a least-square fitting of a straight line and then is subtracted from the data. After that, the original mean value of each time course is added back.

#### 4.5. Bandpass filtering

REST uses an ideal rectangle window to do band-pass filtering as does the equivalent command in AFNI (3dFourier -retrend). The ideal filter transforms the time course into the frequency domain by using the discrete Fourier transform and adds zeros to extend the frequency coverage, then transforms back to the time domain by using the inverse discrete Fourier transform. If the user input for the lower limit of the band range is zero, REST will do a low-pass filtering and, similarly, if the input for higher limit of the band range is greater than the Nyquist frequency, REST will do a high-pass filtering.

#### 4.6. Extract ROI Time Course

This routine is for extracting the mean time course of the voxels within a specified ROI. It could be also used to extract the time course of a single voxel. This utility supports batch mode. The extracted time courses are written as both text files and mat files.

#### 4.7. Regress out covariates

This routine is for regressing out some nuisance covariates. The format for inputting the covariates should be in a text file. The text file must contain one or more columns, each representing a covariate.

#### 4.8. REST Image Calculator

Inspired by the “ImCalc” GUI in SPM and “3dcalc” command line in AFNI, we developed the “REST Image Calculator” to support simple computation on individual images (referred to as i1, i2, i3, …) and group images (g1, g2, g3, …). The expression can be a standard MATLAB expression such as the following examples (a–g) or one of the special functions (h and i):

4.8.1. g1-1: Subtract 1 from each image in group 1 (g1).

4.8.2. g1-g2: Subtract an image in group 2 (g2) from its corresponding image in group 1 (g1).

4.8.3. i1-i2: Subtract an image (i2) from another image (i1).

4.8.4. i1>100: Make a binary mask image at an intensity threshold of 100.

4.8.5. g1>100: Make binary mask images of g1 at an intensity threshold of 100.

4.8.6. g1.* (i1>100): Make a mask image from an image (i1) and then apply it to each image in group 1 (g1).

4.8.7. mean (g1): Generate a mean image of group 1 (g1).

(g) (i1-mean(g1))./std(g1): This example is for the comparison of an individual image with a large group of images, e.g., a patient image with a large group of normal images. The distribution for group 1 (g1) should be normal and the sample size must be large enough (e.g., >100). The resultant Z score map may indicate areas that may be considered to be ‘abnormal’.

4.8.8. corr (g1, g2, “temporal”): Calculate the temporal correlation between 2 4D-images g1 and g2. This can also be used to calculate the correlation between two groups of 3D images, e.g., the correlation between ReHo and ALFF across participants in a voxel-wise way. The output is a correlation coefficient map.

4.8.9. corr (g1, g2, “spatial”): Calculate the spatial correlation between two corresponding 3D images in two groups (number of images ranging from 1 to n). The output is a text file.

#### 4.9. Statistical Analysis

**Figure 6:**
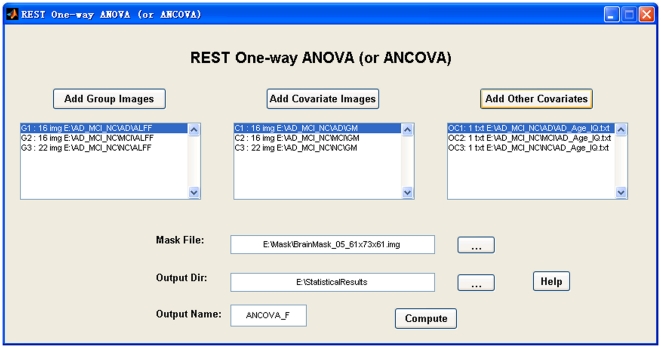
The “REST One-way ANOVA (or ANCOVA)” GUI. This shows an example with covariates (ANCOVA).

**Figure 7:**
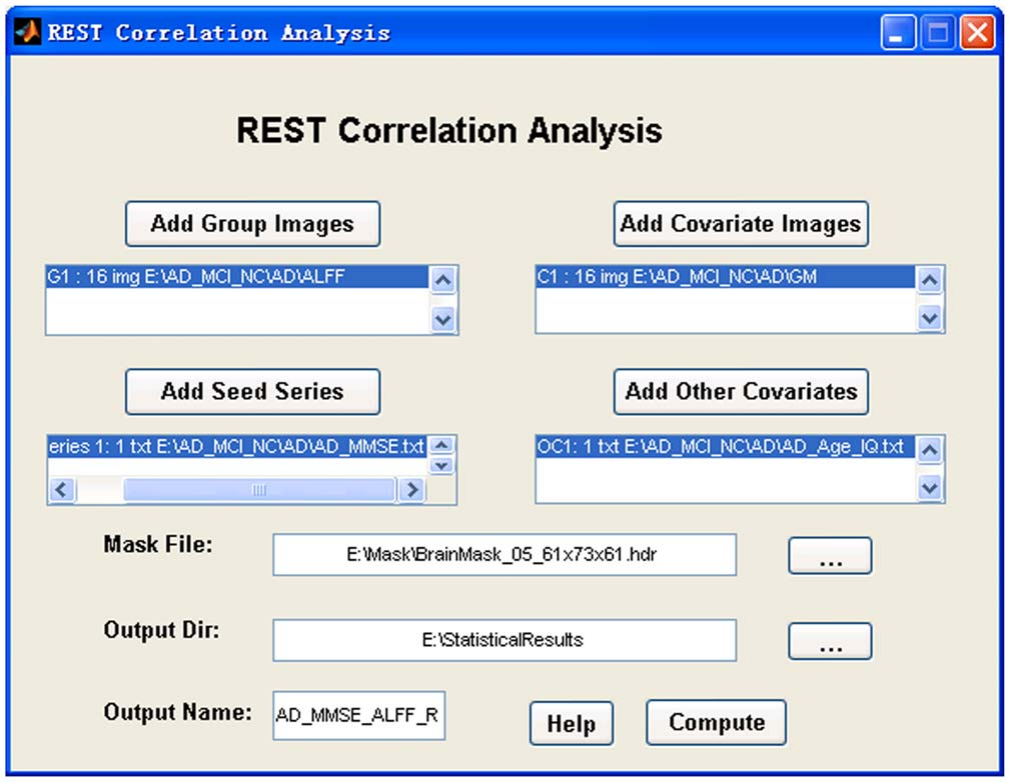
The “REST Correlation Analysis” GUI. This shows an example with covariates.

This section currently supports one-sample T-test, two sample T-test (covariates are optional), paired T-test, one-way ANOVA (or ANCOVA), and linear correlation analysis (covariates are optional). The covariates could be a group of images. For example, the gray matter density can be regressed out in a voxel-wise way when a two-sample T-test is performed between two groups of functional images [48]. The covariates could also be text files. Each text covariate file must correspond to a group of images that are to be compared. The text files (e.g., two text files for two-sample T-test) will be concatenated during analysis. The correlation analysis supports correlation between a text file (e.g., IQ.txt) and a group of images (e.g., ReHo maps of a group of participants). If the covariates are added into the correlation analysis, a partial correlation will be performed. The “Statistical Analysis” portion of REST supports images as covariates in the same way as the function “voxel-dependent EVs” (explainable variables) in FSL (http://www.fmrib.ox.ac.uk/fsl/feat5/detail.html). An example for the “REST One-way ANOVA (or ANCOVA)” GUI is shown in Fig. 6 and the “REST Correlation Analysis” GUI is shown in Fig. 7.

#### 4.10. REST Slice Viewer

**Figure 8:**
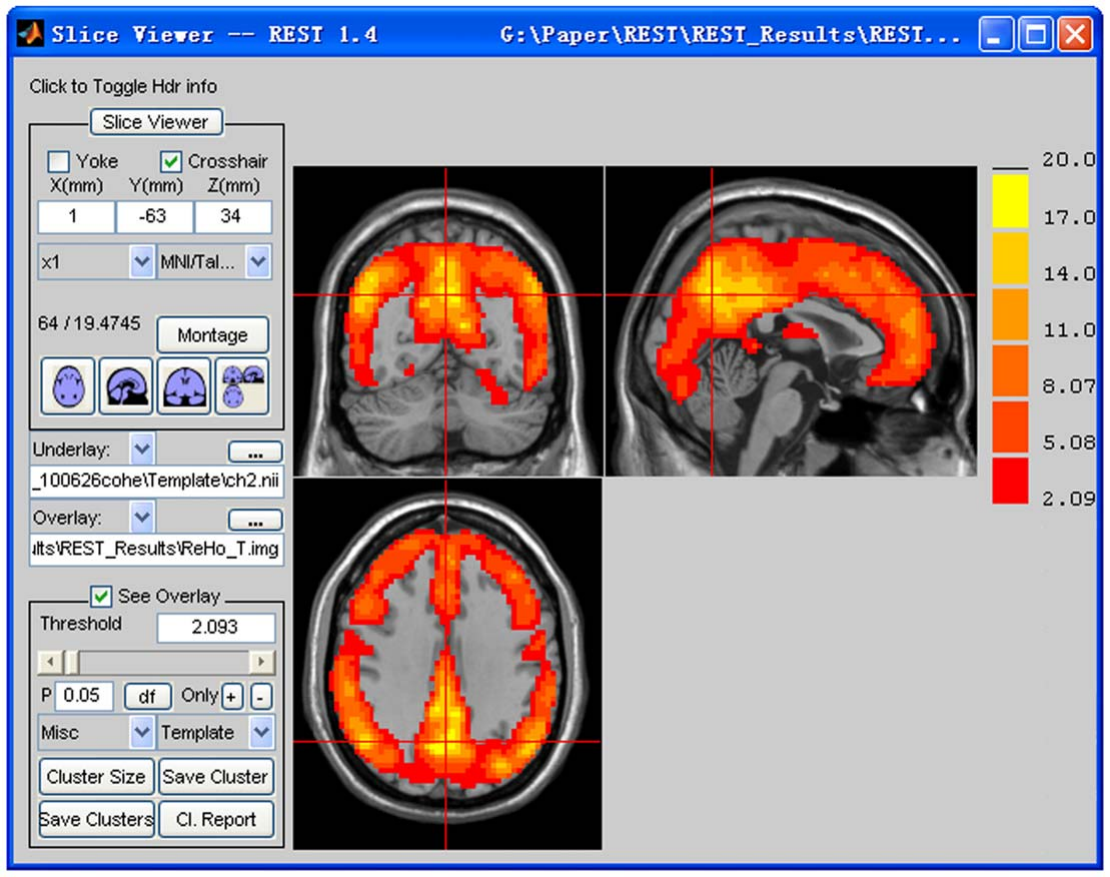
The REST Slice Viewer GUI. The underlay is the Ch2 image (from MRIcro) and the overlay is a result of a T-test (for illustration purpose). The P value is specified as 0.05 and the corresponding T is 2.093. Some common functions are presented by the buttons and other useful functions are listed under the pull-down menu labeled “Misc”.

REST Slice Viewer is a routine for displaying results. The results can be overlaid on a structural image. A color-bar was designed to illustrate the threshold. By clicking the button “Click to Toggle hdr info”, miscellaneous information about the underlay image and the overlay image will be listed, including dimension, voxel size, and origin. It supports some common functions for image-displaying and image-processing, including yoking, montage, orthogonal views, threshold setting, and multiple comparison correction (false discovery rate and AlphaSim under the pulling menu “Misc”). For the false discovery rate, a one-tailed test (like that usually used in SPM, http://www.fil.ion.ucl.ac.uk/spm/) or a two-tailed test can be selected. We recommend using a two-tailed test in most cases. AlphaSim is the same as the command found in AFNI [29] also called ‘AlphaSim’ and is based on a Monte Carlo simulation. “REST Slice Viewer” also reports information about the clusters, including the number of voxels, peak coordinates (in MNI system), anatomical term of location, and peak intensity of each cluster. The cluster report is based on “xjview” (by Xu Cui, http://www.alivelearn.net/xjview/). The REST Slice Viewer GUI is shown in Fig. 8.

#### 4.11. Power Spectrum

**Figure 9:**
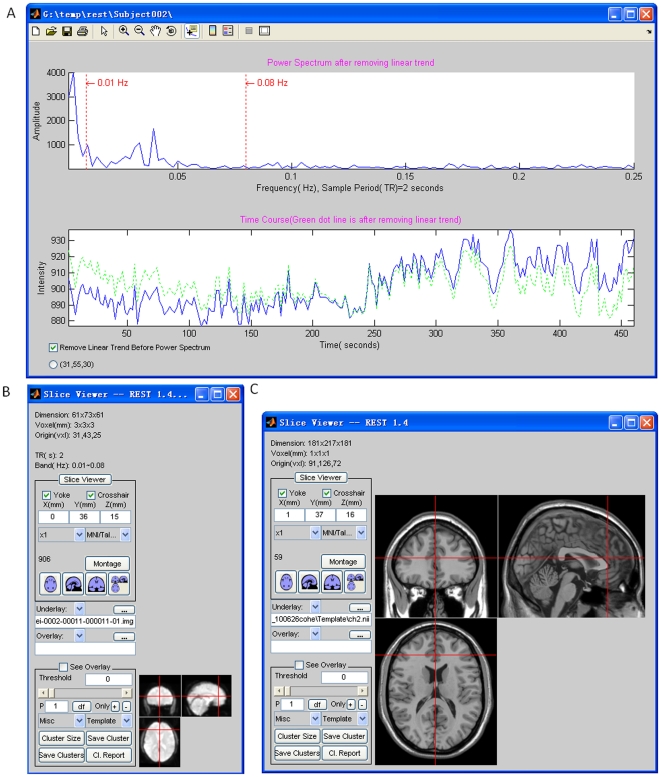
The power spectrum (upper plot in A) and corresponding time course (lower plot in A). Blue line: linear trend not removed; Green line: linear trend removed. The corresponding voxel in the functional image (B), and the corresponding anatomical image (C).

This utility presents a voxel's time course and its power spectrum. It can be used to help check the quality of the fMRI signal. Users need to specify the folder which contains the functional images. As shown in Fig. 9, the time course and the corresponding power spectrum will be shown (Fig. 9A) when a voxel is selected (Fig. 9B). There is an option to remove any linear trend in the time course before calculating the power spectrum.

## Discussion

MATLAB is the runtime engine of REST and therefore some shortcomings of MATLAB, such as inefficient memory management, also exist in REST. Because MATLAB allocates continuous memory for a large matrix, it is a common phenomenon to encounter the “out of memory” error. To reduce the possibility of such a fatal error, REST uses a “space exchanging time” strategy. REST cuts a whole brain 4D dataset into several smaller 4D datasets, and after some memory consuming calculations, REST rebuilds the whole brain 4D dataset. With this strategy, REST can deal with large fMRI dataset with more than 1000 time points on most personal computers.

RS-fMRI studies have provided a few useful computational methods (e.g., functional connectivity, ReHo, and ALFF) for understanding both the physiology and pathophysiology of the spontaneous activity of the human brain. However, the analytic toolkits for RS-fMRI still largely need to be improved. REST was developed as a specialized software package for processing RS-fMRI data. It integrates many calculation methods used in RS-fMRI studies such as functional connectivity, ReHo, and ALFF. It also implements additional functions. REST is an open-source package and provides a framework for implementing more methods. For example, fractional ALFF (fALFF) [38] was implemented in REST v1.3 by Dr. CHENG Wen-lian (See Acknowledgements). A processing pipeline named the Data Processing Assistant for Resting-State fMRI (DPARSF, http://www.restfmri.net, [49]) was implemented based on REST and SPM. A Granger causality analysis package named REST-GCA was developed (Abstract by [50]; See also http://www.restfmri.net). REST is freely available at http://www.restfmri.net. It has been used in RS-fMRI data analysis in a number of recent publications (e.g., [18], [24], [27], [51]–[54]).

### Availability and Future Directions

REST is freely available at http://www.restfmri.net (previously at http://resting-fmri.sourceforge.net before 2008-12-01). The test dataset is available on request. The website http://www.restfmri.net is dedicated to resting-state fMRI research online forum. Users can ask questions and give comments on REST and anything about RS-fMRI. The modular source code analysis of REST based on m2html (http://www.artefact.tk/software/matlab/m2html/, by Guillaume Flandin) is also available (http://restfmri.net/pub/rest_20090422/doc/) to help researchers easily extend REST function or implement new algorithms. Any possible extensions of REST would be welcome and appreciated, since REST is open source in the RS-fMRI community. The full application programming interface (API) could be checked in each REST's script and all the GUI scripts in REST could be regarded as calling samples. Furthermore, DPARSF (http://www.restfmri.net) could be thought as a very good REST's API calling example. ReHo, ALFF/fALFF, functional connectivity computation could be done in scripts like command lines. Some examples could be found in http://restfmri.net/forum/REST_in_scripts.
